# Pediatric Vancomycin Use in 421 Hospitals in the United States, 2008

**DOI:** 10.1371/journal.pone.0043258

**Published:** 2012-08-16

**Authors:** Tamar Lasky, Jay Greenspan, Frank R. Ernst, Liliana Gonzalez

**Affiliations:** 1 MIE Resources, Kingston, Rhode Island, United States of America; 2 Thomas Jefferson University, Nemours Children’s Clinics, Wilmington, Delaware, United States of America; 3 Clinical and Outcomes Research, Premier Research Services, Premier Healthcare Alliance, Charlotte, North Carolina, United States of America; 4 Computer Science and Statistics, University of Rhode Island, Kingston, Rhode Island, United States of America; Columbia University, United States of America

## Abstract

**Background:**

Recommendations to prevent the spread of vancomycin resistance have been in place since 1995 and include guidelines for inpatient pediatric use of vancomycin. The emergence of large databases allows us to describe variation in pediatric vancomycin across hospitals. We analyzed a database with hospitalizations for children under 18 at 421 hospitals in 2008.

**Methodology/Principal Findings:**

The Premier hospital 2008 database, consisting of records for 877,201 pediatric hospitalizations in 421 hospitals, was analyzed. Stratified analyses and logistic mixed effects models were used to calculate the probability of vancomycin use while considering random effects of hospital variation, hospital fixed effects and patient effects, and the hierarchical structure of the data. Most hospitals (221) had fewer than 10 hospitalizations with vancomycin use in the study period, and 47 hospitals reported no vancomycin use in 17,271 pediatric hospitalizations. At the other end of the continuum, 21 hospitals (5.6% of hospitals) each had over 200 hospitalizations with vancomycin use, and together, accounted for more than 50% of the pediatric hospitalizations with vancomycin use. The mixed effects modeling showed hospital variation in the probability of vancomycin use that was statistically significant after controlling for teaching status, urban or rural location, size, region of the country, patient ethnic group, payor status, and APR-mortality and severity codes.

**Conclusions/Significance:**

The number and percentage of pediatric hospitalizations with vancomycin use varied greatly across hospitals and was not explained by hospital or patient characteristics in our logistic models. Public health efforts to reduce vancomycin use should be intensified at hospitals with highest use.

## Introduction

Vancomycin is indicated for the treatment of serious or severe infections caused by susceptible strains of methicillin-resistant (beta-lactam-resistant) staphylococci. It is indicated for patients who cannot receive or who have failed to respond to other drugs, including the penicillins or cephalosporins, and for infections caused by vancomycin-susceptible organisms that are resistant to other antimicrobial drugs. Because of concerns about the development of drug-resistant bacteria, vancomycin should be used only to treat or prevent infections that are proven or strongly suspected to be caused by susceptible bacteria. Recommendations to prevent the spread of vancomycin resistance have been in place since 1995 and include guidelines for inpatient pediatric use of vancomycin [1], [2], [3], [4], [5], [6], [7].

In 1999 Shah and colleagues described vancomycin use in one hospital’s pediatric neurosurgery unit and noted vancomycin was used primarily for prophylaxis and was inconsistent with the Hospital Infection Control Practices Advisory Committee recommendations [8]. Hopkins and colleagues evaluated use in one hospital’s pediatric hematology-oncology unit, and concluded that 100% of the use was not consistent with CDC recommendations [9]. Keyserling and colleagues (2003) studied 22 hospitals belonging to the Pediatric Prevention Network (PPN) [10]. They described series of 25 patients receiving vancomycin at each hospital, and surveyed the physicians who prescribed the vancomycin. They did not categorize adherence to guidelines, but noted general patterns of use such as the low percentage (7%) with laboratory-confirmed β-lactam-resistant organisms isolated at the time vancomycin was prescribed, or the association of vancomycin use with presence of indwelling vascular catheters. Bolon and colleagues evaluated vancomycin use in children older than 1 year at a pediatric tertiary care medical center in 2000 and 2001 [11]. They developed algorithms to evaluate whether use was appropriate, and concluded that 35% of the initial courses were inappropriate. Patel and colleagues studied the medical records of 200 neonates born after Septermber 2005 at 4 tertiary care (NICUs) and concluded that 32% of the days of vancomycin use were inappropriate and non-adherent to the Centers for Disease Control and Prevention 12 Step Campaign to Prevent Antimicrobial Resistance [3], [12]. They noted non-adherence to steps 4 (Target the pathogen) and 8 (Treat infection, not contamination or colonization). Other studies have described use, but have not assessed guideline adherence [13], [14]. The emergence of large multi-hospital databases in the past decade offers new opportunities to study patterns of vancomycin use across hospitals. In this study, we analyzed a large database and describe variation in pediatric vancomycin use in all children under 18 hospitalized at 421 hospitals in 2008.

## Methods

We analyzed vancomycin use occurring in pediatric hospitalizations in 2008 using the Premier hospital database, a large US hospital-based, service-level, all-payer database, containing information from primarily non-profit, non-governmental, community and teaching hospitals and health systems [15]. Detailed service level information was available for each hospital day and included medication information and central supplies. Patient information collected included, but was not limited to, patient demographics (age, gender, race/ethnicity), principal and secondary diagnoses, principal and secondary procedures, payor, length of stay, cost of care, drug utilization, department cost and charge detail, day-of-stay data, and physician specialty. In addition to the service-level data recorded in most standard hospital discharge files, the database provided a daily log of all billed items, including procedures, medications, laboratory tests and diagnostic and therapeutic services, at the individual patient level. Hospitals were self-selected, choosing to provide their own data to Premier as part of agreements by which they received access to analytic tools developed and offered by Premier. In the study year, 2008 423 hospitals participated (99.1% with pediatric hospitalizations). Data were entered into a hospital’s core information system, fed into their decision support system (DSS), then sent to Premier on a monthly or quarterly basis via a secure FTP site. Upon receiving data from participating hospitals, Premier undertook an extensive seven phase data validation and correction process that included more than 95 quality assurance checks. Deidentified data were extracted and used for statistical analysis. The study proposal was approved by the University of Rhode Island Institutional Review Board and informed consent requirements were waived, as permitted under 45 CFR 46.116(c) or (d).

We calculated the number of unique hospitalizations with any use of vancomycin and estimated the prevalence of vancomycin use (number of hospitalizations with documentation of any vancomycin use per 100 hospitalizations). We also estimated the percentages vancomycin use in children over and under 1 year of age, and the percentages of hospitalizations with use longer than 3 days duration. Patient characteristics were: age in years, gender, race (White, African-American, other), and type of insurance (private, government or none). Hospital characteristics were size (small, medium, large), teaching or non-teaching, urban or rural, and region of the country (Northeast, Midwest, South and West, as defined by the US Census).

Logistic mixed effects modeling with two levels of hierarchy (hospital and patient) were used to calculate the probability of vancomycin use while considering the random effects of hospital variation, as well as hospital fixed effects and patient effects. In our models we included the 3 M™ All Patient Refined Diagnosis Related Groups (APR DRGs) severity and mortality (as 4 level, ordered categories), and International Classification of Diseases –9th Revision – Clinical Modification (ICD-9-CM) Group Codes. The 3 M™ APR DRGs expand the basic DRG structure and address patient differences relating to severity of illness and risk of mortality [16]. Severity of illness was defined as the extent of physiologic decompensation or organ system loss of function. Risk of mortality was defined as the likelihood of dying. The SAS procedure, GLIMMIX was run using the events/trial syntax to maintain the hierarchical structure of the data [17], [18], [19]. Models were run in subsets of hospitalizations, for children under 1 year of age, and further separated by ICD-9 Group Codes. Plots of odds ratios were produced using SAS Graphics [20]. All statistical analyses were conducted using SAS 9.2 [21].

**Figure 1:**
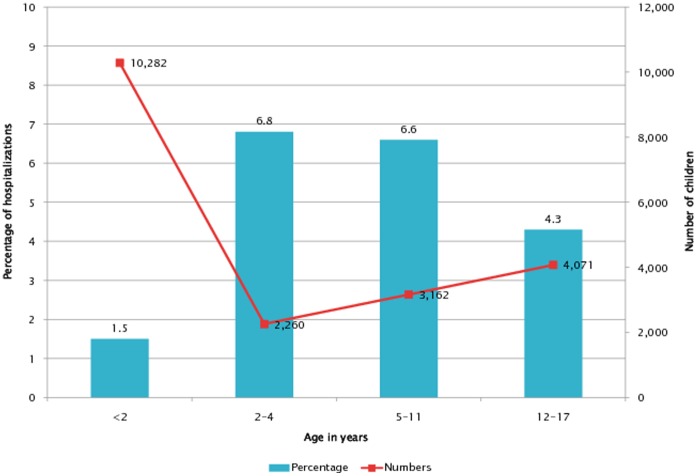
Vancomycin use by age group.

## Results

The dataset contained records for 877,201 hospitalizations of children under 18 at time of admission with 50,879 (5.8%) being repeat admissions. The study population was 50.9% male and 49.1% female, 48.5% white, 16.1% African-American, 12.2% Hispanic, 3.6% Asian/Pacific Islander, 18.7% other and less than 1% American Indian. The average length of stay was 3.7 days (median 2 days, interquartile range 2–3 days). Private insurance paid for 46.2% of the hospital stays, government paid for 45.9% and self-pay, no charge or other sources accounted for 7.9% of the hospital stays. Most of the hospitalizations took place in urban areas (89.2%) compared to rural areas (10.8%).

**Table 1:** The estimates and 95% Confidence Bounds of the variance of the random hospital intercepts on the logit scale in four models for individual diagnostic groups in children under 1.

ICD-group included in model	Estimate of Random Intercept	95% Confidence Bounds
Liveborn infants according to type of birthICD-9-CM V30–39	1.5	1.2–1.9
Infections of skin and subcutaneous tissueICD-9-CM 680–709	1.5	1.1–2.2
Other conditions originating in perinatal periodICD-9-CM 760–779	0.4	0.3–0.7
Congenital anomaliesICD-9-CM 740–759	0.3	0.2–0.9

We validated the Premier sample of hospitalizations by comparing characteristics of the sample to the HCUP KID sample of pediatric hospitalizations for 2006 and details of the validation are available in a previous publication (Lasky et al., 2011). The Premier sample included a greater proportion of infants born in the hospital, from Southern hospitals, from non-teaching hospitals, and from large size hospitals compared to the HCUP KID sample. The two samples were similar with regards to proportions male, routine discharge status, APR-DRGs severity and proportions urban. We did not compare the proportions of different racial and ethnic groups in Premier to the KID because of well documented limitations in racial and ethnic data within the KID, high rates of missing data resulting from state differences in collection and reporting of race and ethnicity. Vancomycin was administered in 19,775 hospitalizations, or 2.3% of 877,201 pediatric hospitalizations in the database in 2008. In 98% of cases, vancomycin was administered parenterally or “other” (this includes ophthalmic solutions, intraocular/intravitreal injections, catheter flushes, inhalation formulations, rectal formulations and topical gels compounded by pharmacy) and in less than 2% of cases, vancomycin was administered orally. Half (10,033 or 50.7%) of the courses of vancomycin were less than 3 days duration. Males had higher prevalence of use than did females (2.5%, 95% CI 2.5–2.6 compared to 2.0%, 95% CI 2.0–2.0), African-Americans had higher use than did whites or other groups (3.1%, 95% CI 3.0–3.2, 2.3%, 95% CI 2.3–2.4 and 1.8%, 95% CI 1.8–1.9 respectively), and children in the age groups 2–4 and 5–11 had higher prevalence compared to children under 2, or children age 12–17 (6.8%, 95% CI 6.5–7.1, 6.6%, 95% CI 6.4–6.8, 1.5%, 95% CI 1.5–1.5, and 4.3%, 95% 4.2–4.4). The greatest number of hospitalizations with vancomycin use occurred to children under 2 (10,282 or 52% of hospitalizations), however the highest prevalence of use occurred in children age 2–4, and 5–11 (Figure 1). Children age 2–4 were 4.5 times more likely, children 5–11 were 4.4 times more likely, and children 12–17 were 2.9 times more likely to receive vancomycin compared to children under 2. In hospitalizations of children under one year with vancomycin use, the four most frequent ICD-9 group diagnoses were: “Liveborn infants according to type of birth” (ICD-9-CM V30–39) (51.83%), “Other conditions originating in the perinatal period” (ICD-9-CM 760–779) (14.29%), “Infections of skin and subcutaneous tissue” (ICD-9-CM 680–709) (6.07%), and “Congenital anomalies” (5.91%) (ICD-9-CM 740–759). In children age 1 or over, the four most frequent diagnoses were: “Infections of skin and subcutaneous tissue” (23.32%), “Pneumonia and influenza”, (9.41%) (ICD-9-CM 480–488), “Complications of surgical and medical care, not elsewhere classifiable” (7.58%) (ICD-9-CM 996–999), and “Other bacterial diseases” (4.41%) (030–041). 

Vancomycin was administered to children at 374 hospitals in the Premier hospital database; another 47 hospitals with 17,271 pediatric hospitalizations (13,233 under age 2) reported no vancomycin use during 2008. The number of hospitalizations with vancomycin use ranged from 0 to 1225 at individual hospitals, and percentage of hospitalizations with vancomycin use ranged from 0.0% to 33.3%. Twenty one hospitals (5.6%) had more than 200 hospitalizations with vancomycin use, accounting for 9,979 (50%) of the pediatric hospitalizations with vancomycin use. Because of the skewness in distribution of vancomycin use, we stratified hospitals by number of hospitalizations with vancomycin use. Low volume was defined as 0 to 10, medium volume as 11–100, and high volume as over 100. Most hospitals were categorized as low volume (221 hospitals), 155 hospitals were categorized as medium volume, and 45 hospitals categorized as high volume. Within high volume hospitals, percentage of hospitalizations with vancomycin use ranged from 1.3–12.9 (mean percentage was 4.6, 95% CI = 3.9–5.4). Within medium volume hospitals, percentage of hospitalizations with vancomycin use ranged from 0.3 to 9.5 (mean percentage was 1.7, 95% CI = 1.5–1.9). The hospitals with high volume of vancomycin use were predominantly large (73.3%), teaching (68.9%), and urban (97.8%) compared to the hospitals with low volume of vancomycin use, which were 45.7% large, 19.91% teaching, and 74.21% urban, and 95% Confidence Intervals of estimates generally did not overlap. In 2008, 47 hospitals, or 11.16% of the hospitals in the database, reported no vancomycin use in the entire year.

**Figure 2:**
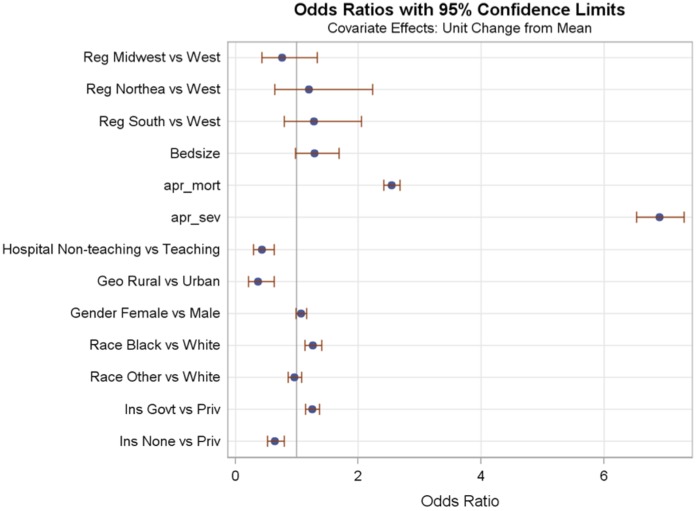
Odds Ratios for models in patients under 1 year of age ICD-9-CM group “Liveborn infants according to type of birth”.

**Figure 3:**
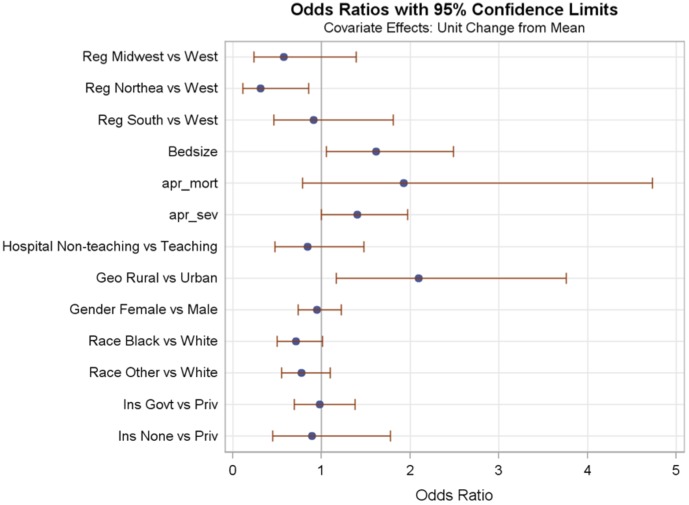
Odds Ratios for models in patients under 1 year of age ICD-9-CM group “Infections of skin and subcutaneous tissue”.

The logistic mixed effects modeling showed hospital variation in vancomycin use. The estimated variance of the random effects hospital intercepts for models run for children under 1, and for the four most frequent ICD-9 Code Groups are summarized in Table 1. The intercept estimates and the 95% Confidence Bounds for each model do not include zero, indicating hospital variation that is statistically significant after controlling for the other variables in the model. The lower limits of the 95 percent confidence intervals are above zero, indicating statistically significant variability in the use of vancomycin depending on the hospital in which a patient was treated.

The Odds Ratios for hospital and patient fixed effects for two of the models are plotted in Figures 2 and 3. Variables associated with vancomycin use were different in each of the age and ICD groups. For example, in children under 1 year with ICD-9 group “Liveborn infants according to type of birth” increased APR-DRG severity of illness was associated with over 6 times the use of vancomycin, but in children under 1 year with ICD-9 group “Infections of skin and subcutaneous tissue” use of vancomycin was almost 2 times as frequent in children with increased APR-DRG severity (although the 95%CI slightly overlapped 1). Both models showed an association between increased APR-DRG severity of illness and vancomycin use, but the magnitude of the effect differed greatly in the two patient groups. Another example is the effect of rural vs. urban status of the hospital. Rural or urban status of the hospital was statistically significant in predicting vancomycin use in children under 1 year with ICD-9 group “Liveborn infants according to type of birth” and with ICD-9 group “Infections of skin and subcutaneous tissue”, but the direction of the effect was different in each group of patients. For children under 1 year with ICD-9 group “Liveborn infants according to type of birth” rural hospitals had lower vancomycin use than did urban hospitals, but for children under 1 year with ICD-9 group “Infections of skin and subcutaneous tissue” rural hospitals had higher vancomycin use than did urban hospitals. Another example can be seen with patient’s insurance coverage. In children under 1 year with ICD-9 group “Liveborn infants according to type of birth” those with government insurance had slightly higher vancomycin use than did those with private insurance, and those with no insurance had less vancomycin use than those with private insurance. In children under 1 year with ICD-9 group “Infections of skin and subcutaneous tissue” insurance coverage was not associated with vancomycin use.

## Discussion

Most hospitals (221) had fewer than 10 hospitalizations with vancomycin use in the study period, and 47 hospitals reported no vancomycin use in 17,271 pediatric hospitalizations. At the other end of the continuum, 21 hospitals (5.6% of hospitals) each had over 200 hospitalizations with vancomycin use, and together, accounted for more than 50% of the pediatric hospitalizations with vancomycin use. Percentage of hospitalizations with vancomycin use ranged up to 33.3% when hospitals with few pediatric hospitalizations were kept in the sample, and for this reason, percentage, by itself, may not be a useful indicator in small hospitals. In hospitals with more than 100 hospitalizations with vancomycin use, the percentage with vancomycin use ranged from 1.26 to 12.90, a 10 fold range in the probability of vancomycin use. Without knowing how percentage correlates with inappropriate use, one might begin by evaluating vancomycin use in hospitals with greater than 100 hospitalizations with vancomycin use and greater than 4 or 5% of hospitalizations having vancomycin. The two measures, absolute number of hospitalizations and percentage of hospitalizations with vancomycin use, can be used to identify and target hospitals for evaluation and potential interventions.

The limitations of the study include those inherent in secondary analysis of large databases. In this database, the ICD-9 code referred to the entire hospitalization rather than the indication for medication use, and we were unable to analyze the relationship between Methicillen-resistant Staphylococcus aureus (MRSA) occurrence and vancomycin use. The Premier database codes for MRSA changed during 2008 and future analyses will be able to describe MRSA variation. Although we could not analyze the data from our study year, 2008, a preliminary analysis of 2011 data showed MRSA codes in only 9.6% of hospitalizations with vancomycin use. Future analyses whether this is explained by undercoding MRSA, inappropriate use of vancomycin, or some other difference.

The definition of vancomycin use as any use during the hospital stay is both a weakness and a strength. It does not permit analysis of dose or duration which will be of interest in future studies, but it does permit estimates of percentages of hospitalized children exposed to vancomycin use. This is of interest when planning clinical studies, comparative effectiveness research, policy and labeling priorities, and other issues. The percentage (or prevalence) may provide a different perspective than that developed as a result of using numerator data only. For example, Keyserling and colleagues (2003) tabulated vancomycin courses and identified the largest number as occurring in neonatology services, and then suggested that neonatal intensive care units (NICUs) improve their vancomycin use [10]. We also found the highest number of uses in children under 2 years of age, but the lowest probability of use in children under 2 years of age (1.5%) compared to other age groups (4.3–6.8%). This may reflect the large number of healthy newborns in databases and programs to measure use of vancomycin in neonates will need to define a denominator, in addition to measuring the numerator, as is currently done.

The stratified analysis and the logistic modeling consistently document variation in vancomycin use by individual hospital, after considering independent effects of hospital and patient characteristics. The mixed models allowed us to estimate variation in the use of vancomycin by hospital in children with the same ICD-9 group codes. The intercepts measure the differences between hospitals, controlling for other effects in the model such as hospital and patient characteristics [18]. For every ICD-9 Group modeled, hospital-to-hospital variation and 95% Confidence Bounds of the intercept excluded zero, after controlling for hospital and patient effects.

Until recently, few studies have compared antibiotic use across large numbers of hospitals or geography, however the establishment of the National Healthcare Safety Network by the Centers for Disease Control and Prevention will allow regular comparisons across hospitals [22]. Geographic variation in use of antibiotics has also recently been documented in the United States [23].

The analyses presented here are also a step forward in studying pediatric hospital variation; previous researchers have used hierarchical models to consider hospital variation in adults, for example by studying maternity length of stay or mortality in patients undergoing coronary artery bypass surgery (CABG) [18], [24]. Hospital variation in care of adults has been studied for several decades, much of it made possible by large Medicare claims databases [25]. Only recently have aggregated data for pediatric hospitalizations been available. Bowman and colleagues (2005) recently demonstrated variation in management of pediatric splenic injuries and Weiss et al (2009) have demonstrated variation in use of corticosteroids, opioids and nonsteroidal anti-inflammatory drug, diagnostic imaging, and renal biopsy in children with Henoch Schönlein purpura [26], [27]. The public health implications of these data are that efforts to control vancomycin use may be channeledo hospitals with high numbers and prevalence of vancomycin use. While it seems intuitive that high prevalence will lead to high volume, and vice versa, some high volume hospitals maintain a prevalence below 3 per cent, while others range above 7 percent, almost to 13 percent. Presently, the message to reduce vancomycin use is broadcast to the entire healthcare community [2], [28]. General principles of judicious antibiotic use are applicable to all hospitals and providers for all antibiotics, and these data may be used to channel intensive stewardship activities and intervention research data to hospitals with the highest volume and prevalence of vancomycin use.

While the Premier hospital database contains information about presence or absence of laboratory testing, it does not include information about test results. Future research relating vancomycin use to laboratory test results may need to budget for and obtain access to hospital charts or to laboratory-based infection surveillance data. We restricted the current analysis to one year, 2008; access to other years of data would permit assessment of trends over the last several years. Finally, further study may prepare the way for comparative effectiveness research, by identifying and comparing children with similar conditions treated with and without vancomycin, and relating the treatment patterns to outcomes.

Our key findings include the skewness of the distribution of vancomycin use throughout hospitals, the importance of denominator data in assessing vancomycin use, and hospital variation in vancomycin use, not explained by hospital or patient characteristics including: bed size, teaching status, region of the country, rural or urban geography, and patient sex, race, APR-DRG risk of mortality and APR-DRG severity of illness.
